# Long non-coding RNA BC087858 induces non-T790M mutation acquired resistance to EGFR-TKIs by activating PI3K/AKT and MEK/ERK pathways and EMT in non-small-cell lung cancer

**DOI:** 10.18632/oncotarget.10521

**Published:** 2016-07-09

**Authors:** Hui Pan, Tao Jiang, Ningning Cheng, Qi Wang, Shengxiang Ren, Xuefei Li, Chao Zhao, Limin Zhang, Weijing Cai, Caicun Zhou

**Affiliations:** ^1^ Department of Medical Oncology, Shanghai Pulmonary Hospital, Tongji University School of Medicine, Yangpu District, Shanghai, China; ^2^ Department of Lung Cancer and Immunology, Shanghai Pulmonary Hospital, Tongji University, Tongji University Medical School Cancer Institute, Shanghai, P. R. China

**Keywords:** long non-coding RNA, non-small-cell lung cancer, non-T790M mutation, acquired resistance, EGFR-TKIs

## Abstract

Our previous study demonstrated that long non-coding RNA (lncRNA) BC087858 could stimulate acquired resistance to EGFR-TKIs in non-small cell lung (NSCLC) but the specific regulatory mechanism remained unknown. We aimed to explore the role and mechanism of lncRNA BC087858 on EGFR-TKIs acquired resistance. LncRNA BC087858 mRNA expression was detected by reverse transcription polymerase chain reaction in different NSCLC cell lines and tissues. The relationship between BC087858 expression and clinicopathological factors was performed by Cox multivariate regression analysis. Small-interfering RNA, flow cytometry and trans-well assay were conducted to explore the biological functions of BC087858. Western blotting was used to analyze the target proteins expression. Over-expression was observed in NSCLC cells and patients with acquired resistance to EGFR-TKIs and significantly associated with a shorter progression-free survival (PFS) (12.0 vs. 17.0 months, *P* = 0.0217) in tumors with respond to EGFR-TKIs. The significant relationship was not observed in patients with T790M mutation (median PFS 17.6 vs. 12.5 months, *P* = 0.522) but in patients with non-T790M (median PFS 8.0 vs. 18.25 months,*P* = 0.0427). Down-regulation of BC087858 could significantly promote PC9/R and PC9/G2 cells invasion (*P* < 0.05; respectively). BC087858 knockdown restored gefitinib sensitivity in acquired resistant cells with non-T790M and inhibited the activation of the PI3K/AKT and MEK/ERK pathways and epithelial-mesenchymal transition (EMT) via up- regulating ZEB1 and Snail. In conclusion, LncRNA BC087858 could promote cells invasion and induce non-T790M mutation acquired resistance to EGFR-TKIs by activating PI3K/AKT and MEK/ERK pathways and EMT via up- regulating ZEB1 and Snail in NSCLC.

## INTRODUCTION

Lung cancer is the leading cause of cancer-related mortality accounting for an estimated 1.59 millions deaths worldwide [1, 2]. More than 85% of those cases currently classified as non-small-cell lung cancer (NSCLC) [3]. Discovery of activating mutations of epidermal growth factor receptor (EGFR) and their use as predictive biomarkers to tailor patient treatment with EGFR tyrosine kinase inhibitors (TKIs) has revolutionized therapy of patients with advanced EGFR-mutant NSCLC [4]. However, despite an initial response, the median progression-free survival (PFS) of patients treated with EGFR-TKIs limits from 10 to 14 months due to drug resistance, also named acquired resistance, which remains a major problem in clinic [5–8]. The most common mechanism whereby acquired resistance to EGFR-TKIs develops is a secondary T790M mutation (> 50–60% of patient cases) [9]. As well as this mechanism, c-MET amplification (5– 10%), HER2 amplification (12%), PIK3CA mutations (5%), BRAF mutations (1%), small-cell lung cancer histological transformation (3–14%) and so on are also associated with acquired resistance to EGFR-TKIs [4]. However, the mechanisms responsible for about 20–30% of cases of acquired resistance to EGFR-TKIs are still unknown [10].

Long non-coding RNAs (lncRNAs) are usually described as a group of non-coding RNA of longer than 200 nucleotides that are involved in multiple molecular genetic and cellular processes including cell apoptosis, tumor invasion, migration, metastasis, and drug resistance [11–14]. Various studies have suggested that lncRNAs, including UCA1, HOTAIR, H19, CUDR, AK126698 and MALAT1 are related to chemotherapy and/or EGFR-TKIs resistance [11, 14–17]. In our previous study, we compared the expression of lncRNAs in gefitinib-sensitive and gefitinib-resistant human lung cancer cells by lncRNA microarray analysis, and found that some lncRNAs, including lncRNA BC087858, were up-regulated in resistant cells [18].

In the present study, we aimed to determine the biological function of lncRNA BC087858 and whether it could induce acquired resistance to EGFR-TKIs by activating PI3K/AKT/ERK pathway and epithelial-mesenchymal transition (EMT) in EGFR-mutant NSCLC.

## RESULTS

### Over-expression of BC087858 was correlated with acquired resistance to EGFR-TKIs

**Figure 1:**
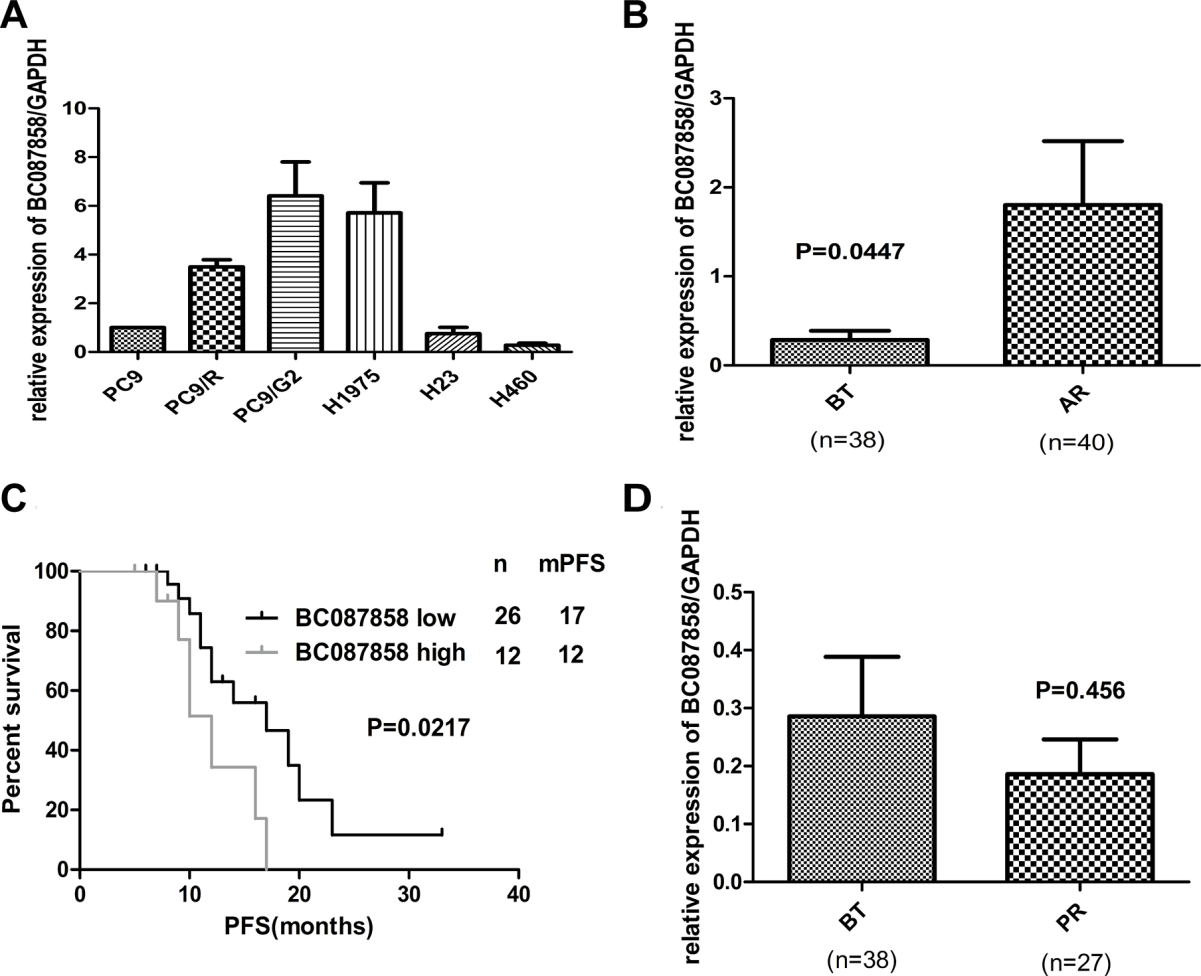
(A) The expression of BC087858 in lung cancer cells. Over-expression of BC087858 was observed in lung cancer cells with acquired resistance (H1975, PC9/R and PC9/G2 cells) but not found in primary resistant cells (H23, H460) compared to sensitive cell line(PC9). (**B**) BC087858 expression levels in lung cancer tissues assessed by qRT-PCR in patients with *EGFR*-TKI-sensitive NSCLC (BT group) and patients who developed acquired resistance to EGFR-TKIs(AR group). (**C**) Progression-free survival (PFS) in patients with high and low BC087858 expression levels before *EGFR*-TKI treatment. (**D**) BC087858 expression levels were assessed in patients before *EGFR*-TKIs treatment and primary resistance subgroup. BT: before treatment; AR: acquired resistance; PR: primary resistance; PFS: progression-free survival.

To unveil the mechanisms of acquired resistance to EGFR-TKIs, we performed microarray expression profiling of lncRNAs/mRNA for PC9 and PC9/R cells. BC087858 was found to have a high expression level in PC9/R cells with acquired resistance to gefitinib [18]. To validate the analysis of lncRNAs profiles, we assessed the mRNA expression of BC087858 by qRT-PCR in different lung cancer cell lines and tissues from patients before EGFR TKIs treatment (BT group) and after harboring acquired resistance to TKIs (AR group). The clinical information of the two groups was shown in Table 1. We also added 27 patients who were defined as primary resistance. In NSCLC cell lines, BC087858 was higher in TKIs acquired resistance cells (H1975, PC9/R and PC9/G2) than primary resistant cell lines (H23 and H460) and TKIs sensitive cell line PC9 (Figure 1A). BC087858 mRNA expression level in patients who developed acquired resistance to EGFR-TKIs was significantly higher than in patients before TKIs treatment (1.802 ± 0.7175 vs. 0.2855 ± 0.1029, *P* = 0.0447; Figure 1B). On the basis of the BC087858 expression before treatment with EGFR-TKIs, the patients were divided into a high expression group (*n* = 26) and a low expression group (*n* = 12), depending on whether they were above or below the cut-off value 2^-ΔCt^ = 0.142 (Supplementary Table 1). When progression free survival (PFS) was assessed, patients in high-BC087858 group had a significantly poorer prognosis than low-BC087858 group (median PFS 12.0 vs. 17.0 months, *P* = 0.021; Figure 1C). The patients with primary resistance to TKIs (*n* = 27) had a lower expression of BC087858 than patients before TKIs treatment but not significant (0.1862 ± 0.05987 vs. 0.2855 ± 0.1029, *P* = 0.4599; Figure 1D). Univariate analysis of PFS revealed that the expression level of BC087858 EGFR mutation types and age were prognostic indicators, while multivariate analysis indicated that EGFR mutation types and age were independent prognostic factors for PFS in patients with EGFR-TKI-sensitive NSCLC. The BC087858 expression level was also associated with prognosis but it just reached the marginal statistical significance (*P* = 0.083; Table 2).

**Table 1:** Clinical characteristics of 38 NSCLC patients with EGFR-mutantation (BT group) and 40 with aquired resistance to EGFR-TKIs (AR group)

clinical characteristics	BT group *N* = 38	AR group *N* = 40
Age		
< 65	27 (71.0%)	28 (70.0%)
≥ 65	11 (29.0%)	12 (30.0%)
Gender		
Male	13 (34.2%)	21 (52.5%)
Female	25 (65.8%)	19 (47.5%)
Distal metastasis		
M0	2 (5.2%)	7 (17.5%)
M1	36 (94.8%)	33 (82.5%)
TNM Stage		
III b	2 (5.2%)	8 (20.0%)
IV	36 (94.8%)	32 (80.0%)
EGFR mutation		
19DEL	24 (63.1%)	18 (45.0%)
L858R	16 (42.1%)	9 (22.5%)
T790M	2 (5.2%)	21 (52.5%)
Smoking		
non-smoker	31 (81.6%)	24 (60.0%)
smoker	7 (18.4%)	16 (40.0%)
TKIs		
Gefitinib	28 (73.7%)	15 (37.5%)
Erlotinib	10 (26.3%)	25 (62.5%)
Histologic type		
Adenocarcinoma	32 (84.2%)	35 (87.5%)
squamous carcinoma	0 (0.0%)	1 (2.5%)
adeno-squamous carcinoma	1 (2.6%)	2 (5.0%)
NSCLC	5 (13.2%)	2 (5.0%)
BC087858		
low	26 (68.4%)	24 (60.0%)
high	12 (31.6%)	16 (40.0%)

**Table 2:** Univariate and multivariate analysis for progression-free survival (PFS)

Factors	Univariate analysis		Multivariate analysis	
	HR (95% CI)	*P*	HR (95% CI)	*P*
Age (< 65/≥ 65 years)	0.392 (0.128–1.197)	0.1	0.199 (0.047–0.849)	0.029
Sex (male/female)	1.649 (0.626–4.347)	0.312		
Smoking (never/ever)	1.576 (0.432–5.752)	0.491		
EGFR (19DEL/L858R)	0.289 (0.104–0.804)	0.017	0.217 (0.053–0.896)	0.035
Histology(adenocarcinoma/non-adenocarcinoma)	0.626 (0.139–2.815)	0.542		
Stage (IV/IIIB)	1.826 (0.231–14.438)	0.568		
BC087858 (low/high)	2.781 (1.022–7.569)	0.045	2.51 (0.888–7.097)	0.083
EGFR-TKIs (gefitinib/erlotinib)	0.482 (0.147–1.577)	0.228		

CI: confidence interval; EGFR: epidermal growth factor receptor; HR: hazard ratio;

TKI: tyrosine kinase inhibitor;

### The effect of over-expression of BC087858 on PFS for patients with acquired resistance to EGFR-TKIs was from T790M-negative subgroup

**Figure 2:**
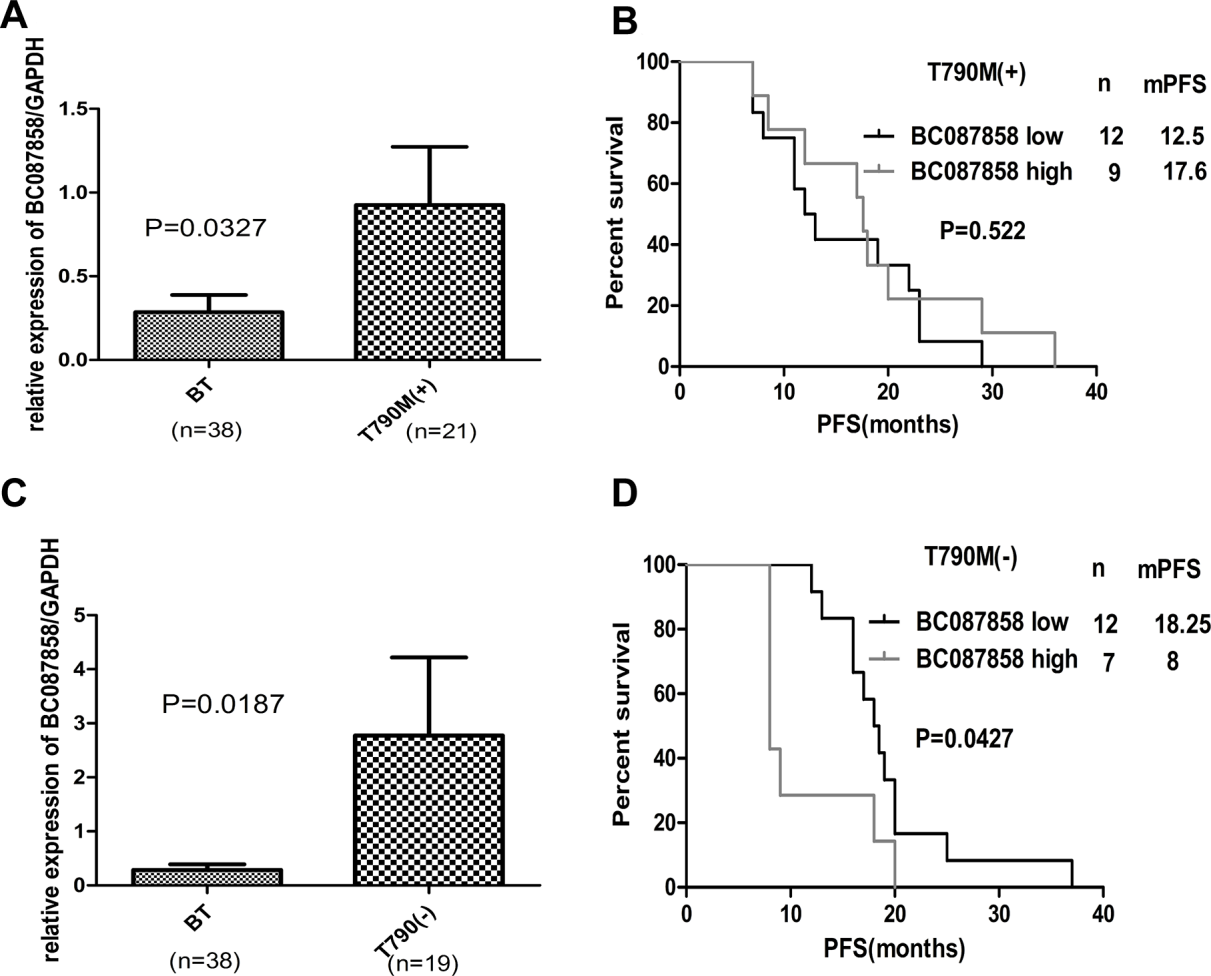
(**A–C**) expression levels of BC087858 assessed in patients treated with EGFR-TKI-sensitive NSCLC (BT group) and patients who were with and without T790M mutations. (**B–D**) PFS in patients with acquired resistant who were with T790M mutations and without T790M mutations.

To explore the mechanism of BC087858 in TKIs required resistance, the AR group was departed as two groups due to whether they harbored T790 mutation. Compared with patients before TKIs treatment, BC087858 expressed higher in T790M positive (0.2855 ± 0.1029 vs. 0.9253 ± 0.3482, *P* = 0.0327 Figure 2A) and T790M negative groups (0.2855 ± 0.1029 vs. 2.772 ± 1.1449, *P* = 0.0187; Figure 2C). When examined the correlation of BC087858 over-expression on PFS, no relationships were found in T790M positive subgroup (median PFS 17.6 vs. 12.5 months, *P* = 0.522; Figure 2B), in T790M negative subgroup significant association were found (median PFS 8.0 vs. 18.25 months, *P* = 0.0427; Figure 2D). Therefore, we hypothesized that BC087858 might play a critical role in acquired resistance to EGFR-TKIs in patients without T790M mutation.

### Down-regulated BC087858 partially restored gefitinib sensitivity *in vitro*

**Figure 3:**
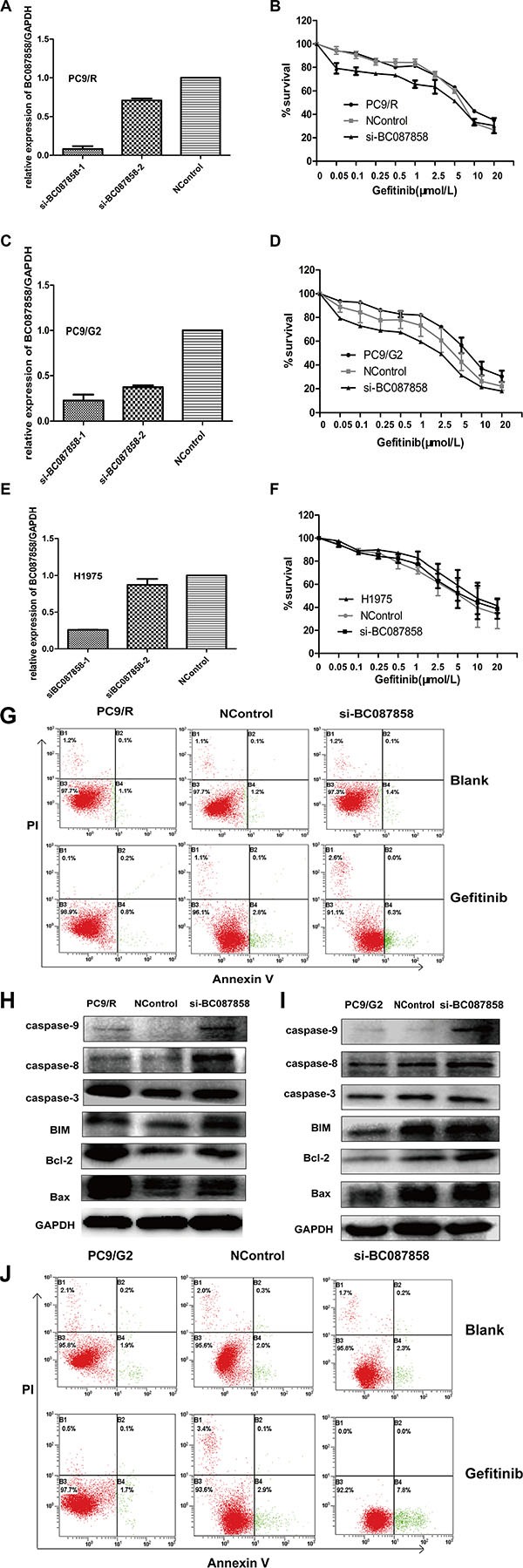
(**A–E**) qRT-PCR detection of BC087858 expression in PC9/R, PC9/G2 and H1975 cells after silencing of BC087858 by si-RNA. The relative expression of BC087858 was at least 60% lower with the negative control. (**F**) The sensitivity to gefitinib of PC9/R, PC9R/G2 and H1975 cells was detected by CCK-8 (Cell Counting Kit-8). Cells were exposed to various concentration of gefitinib for 72 hours. Inhibiting the BC087858 gene resulted in an approximately 2-fold decrease in the gefitinib IC_50_ in PC9/R cells (IC_50_ in si-BC087858-PC9/R and PC9/R cells,4 μmol/L and 9 μmol/L, respectively), and the IC50 in PC9/G2 cells was also lower down. (**G–J**) Gefitinib-induced apoptosis in PC9/R and PC9/G2 cells was demonstrated by flow cytometric analysis. Cells were treated with gefitinib for 72 hours and then analyzed for early apoptotic cells (bottom right quadrant) and late apoptotic cells (top right quadrant). The percentages of cells in the two quadrants are shown. H.,I. caspase 9, caspase 3, caspase 8, Bcl-2 and BIM(Bcl-2 interacting mediator of cell death), Bax(co-mediator of cell death).

To test the role of BC087858 in NSCLC cells, siRNA was transfected in TKIs acquired resistance cell lines PC9/R, PC9/G2 and H1975. The expression of BC087858 were down-regulated 60% to 90% using si-BC087858-1 (Figure 3A, 3C, 3E). Down-regulated BC087858 partially reduced the resistance to gefitinib in PC9/R and PC9/G2 cells compared to blank and negative controlled cells (Figure 3B, 3D, 3F). However, these changes were not observed in T790 mutation cell line H1975. These results conformed to the clinical data. To further identify the mechanism of down-regulated BC087858 in PC9/R and PC9/G2 cell lines, apoptosis cell rate were assessed after 72 hours treated with 5 μmol/L gefitinib. Down regulated BC087858 increased two-fold of the apoptosis cells both in PC9/R and PC9/G2 cell lines (Figure 3G, 3J).

### BC087858 enhanced cell invasion

**Figure 4:**
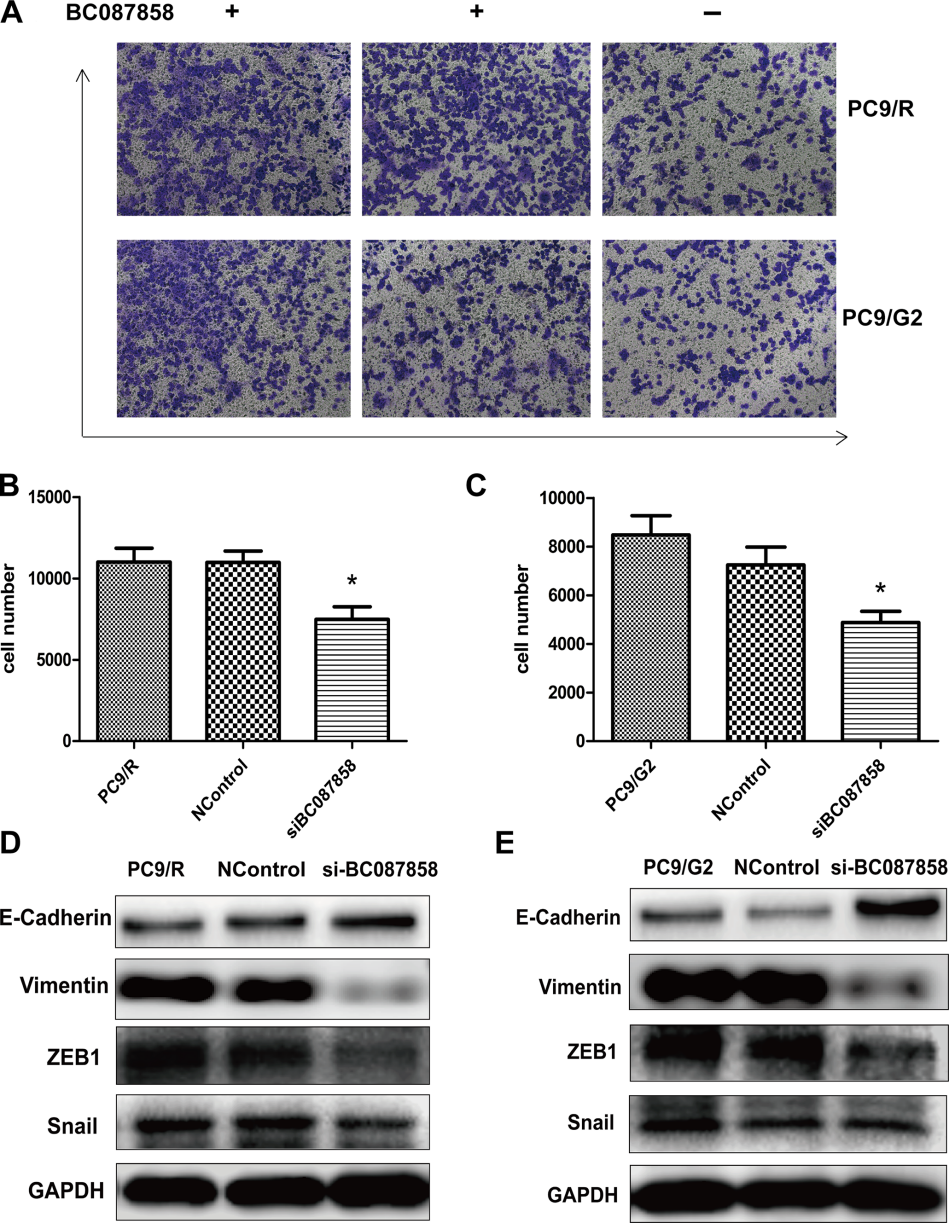
(A) The *in vitro* cell invasion of PC9/R and PC9/G2 cell after silencing of BC087858 by si-RNA (**B–C**) 5 migration cell number were counted 5 visions per well. (**D–E**) E-cadherin, Vimentin, Snail and ZEB1(co-mediator of EMT). EMT: epithelial-mesenchymal transition.

To explore the correlation of BC087858 on invasion ability in acquired resistance lung cancer cell, we conducted knockdown of over expressed BC087858 with specific siRNA to stably BC087858-expressing PC9/R and PC9/G2 cells and trans-well assay. The results showed that down-regulation of BC087858 could significantly promote PC9/R and PC9/G2 cells invasion (*P* < 0.05; respectively; Figure 4A–4C).

### BC087858 may promote activation of PI3K/AKT pathway and EMT through up-regulating Snail and ZEB1

**Figure 5:**
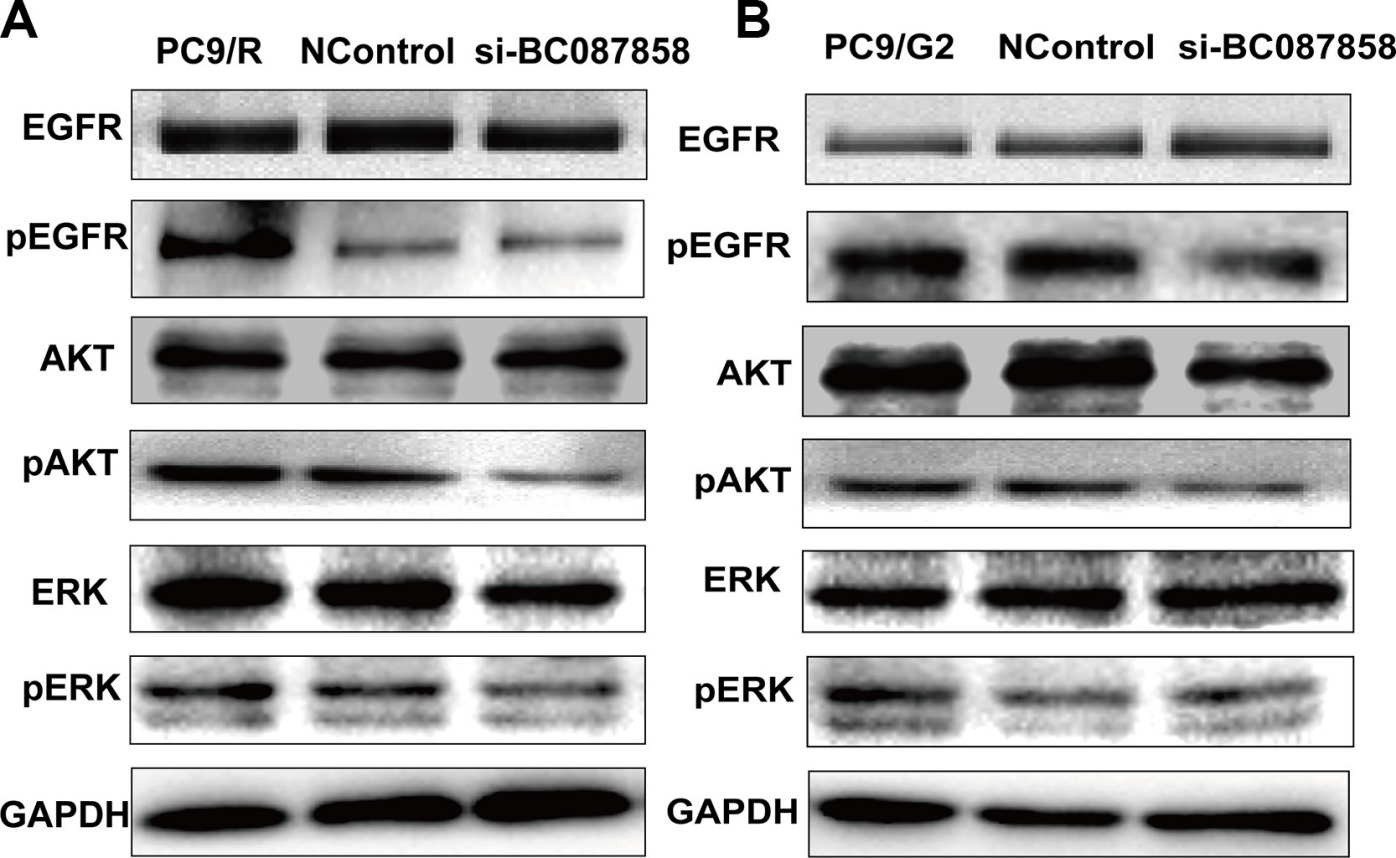
(A–B) Effect of BC087858 on the activity of the PI3K/AKT and MEK/ERK pathway

To further explore the molecular mechanisms of BC087858 in acquired resistance to EGFR-TKIs, we assessed the correlation between BC087858 and crucial proteins functioned in signaling pathways. Western blot analysis showed that E-cadherin was up-regulated, while Vimentin, ZEB1 and Snail were down-regulated when knockdown of BC087858 expression (Figure 4D, 4E). These results indicated that over-expression of BC087858 might promote activation of EMT through up-regulating Snail and ZEB1. Furthermore, down-regulation of BC087858 inhibited the protein levels of phospho-EGFR and the downstream signaling proteins phospho-AKT and phospho-ERK (Figure 5A, 5B) compared with resistant and normal control cell lines. Collectively, these results suggested that over-expression of BC087858 activate PI3K/AKT and MEK/ERK pathway and EMT via up-regulating Snail and ZEB1 to promote resistance to EGFR-TKIs.

## DISCUSSION

EGFR-mutant NSCLC patients who benefited from EGFR-TKI eventually develop acquire resistance to these therapies [4, 19]. Although a number of studies revealed that a variety of mechanisms can stimulate acquired resistance to EGFR-TKI including secondary mutations within EGFR at position T790, mutation in EGFR effector proteins, small-cell lung cancer histologic transformation and upregulation of parallel receptor tyrosine kinases (e.g., MET, HER2 and AXL) [4], the mechanisms responsible for about 20–30% of cases of acquired resistance to EGFR-TKIs are still unknown. Moreover, the mechanisms responsible for most patients with non-T790M acquired resistance to EGFR-TKIs are also unknown [10]. Our research team has been working on the epigenetics especial non-coding RNAs which have been revealed played a key role in EGFR-TKIs resistance [20]. In our previous studies, we reported that miR-21, miR-214 and miR-200c are involved in both acquired resistance and primary resistance to EGFR-TKIs [21–23]. However, lncRNAs can connect to transcription sites and regulate both the expression of alleles and a long fragment, whereas coding genes and micro-RNAs have no such functions, thus suggests that lncRNAs may be better epigenetic regulators in controlling performance. We have identified some lncRNAs including lncRNA UCA1, H19, BC200 and BC087858 were up-regulated in gefitinib-resistant human lung cancer cells by lncRNA microarray analysis [18]. Also we have demonstrated that lncRNA UCA1 may induce non-T790M acquired resistance to EGFR-TKIs by activating the AKT/mTOR pathway and EMT [24]. However, the role and mechanisms of lncRNA BC087858 in EGFR-TKIs acquired resistance remain unknown. Therefore, we performed this study to speculate the role and possible molecular mechanism of lncRNA BC087858 in acquired resistance to EGFR-TKIs in NSCLC.

To our knowledge, this is the first study to explore the role lncRNA BC087858 expression in EGFR-TKIs acquired resistance. In the present study, we found that over-expression of lncRNA BC087858 was associated with acquired resistance to EGFR-TKIs both in NSCLC cell lines and patients. Our clinical data indicated that lncRNA BC087858 expression levels were significantly higher in EGFR-mutant NSCLC patients who developed acquired resistance to EGFR-TKIs compared with before treatment levels. This result suggested that high expression of lncRNA BC087858 could play a crucial role in the resistance to EGFR-TKIs. In addition, over-expression of lncRNA BC087858 was correlated with the poorer prognosis than those in the low expression group. The patients with primary resistance to TKIs had a lower expression of BC087858 than patients before TKIs treatment. Univariate and multivariate analysis of PFS revealed that lncRNA BC087858, EGFR mutation types and age were independent prognostic factors. Of note, we found that over-expression lncRNA BC087858 was not significantly associated with PFS for patients with T790M acquired resistance to EGFR-TKIs, although the significant high expression level of lncRNA BC087858 in NSCLC with acquired resistance regardless of T790M status was observed. Hence, we hypothesized that high expression of lncRNA BC087858 may be one of the mechanisms of acquired resistance to EGFR-TKIs in EGFR-mutant NSCLC without T790M. This hypothesis was demonstrated by our *in vitro* study that lncRNA BC087858 knockdown could partly restore the sensitivity of PC9/R and PC9/G2 cells (19DEL, without T790M or MET amplification).

The present study also revealed that lncRNA BC087858-mediated acquired resistance to gefitinib may occur through activation of the PI3K/AKT and MEK/ERK pathways and EMT via up-regulating ZEB1 and Snail. Previous studies have implicated activation of the PI3K/AKT and MEK/ERK pathways as well as EMT in resistance to EGFR-TKIs [25, 26]. Previous studies have showed that activation of EMT can induced E-cadherin repression and directly deduce EGFR-TKIs sensitivity. EMT activation also leads to higher prevalence of the EGFR T790M mutated allele. However, combined inhibition of EGFR and EMT was not sufficient to prevent acquired gefitinib resistance because of an increased emergence of the EGFR T790M allele compared with cells treated with gefitinib alone. Thus reveals that EMT may induce acquired EGFR-TKIs resistance especially in non-T790M mutation cases [27–29]. Subsequently, other studies have reported that lncRNA BC087858 can be located near forehead box protein C1 (FOXC1), which is a member of the FOX transcription factor family and is important in cancer development. FOXC1 induces EMT through inhibition of E-cadherin expression and promotes cell migration and invasion in hepatocellular carcinoma [30]. These studies confirm the validity of our results. Moreover, out study also found that up-regulation of ZEB1 and Snail instead of FOXC1 over-expression was the reason of lncRNA BC087858-activated EMT in NSCLC. Taken together, we considered lncRNA BC087858 may be associated with PI3K/AKT and MEK/ERK pathways and EMT. Further investigations will be required to elucidate the mechanisms by which lncRNA BC087858 regulates the PI3K/AKT and MEK/ERK signaling pathways.

In recent years, third generation EGFR-TKIs such as CO-1686 and AZD9291 that overcome acquired resistance caused by T790M have showed promising and amazing results in the phase 1 studies and the phase 3 studies are ongoing [31, 32]. To date, however, patients without T790M mutations who develop acquired resistance to EGFR-TKIs have no effective treatment, as the mechanisms of acquired resistance remain unclear. Some studies have reported that the histone lysine-specific demethylase 1 (LSD1) enzyme EZH2 may be a new “druggable” epigenetic target [33–35]. Therefore, we consider that lncRNA BC087858 may play a significant role in overcoming non-T790M acquired resistance to EGFR-TKIs by functioning as a new epigenetic regulator in NSCLC.

In conclusion, we have defined that lncRNA BC087858 over-expression was significantly associated with poor prognosis of NSCLC patients with acquired resistance to EGFR-TKIs and the correlation of over-expression of lncRNA BC087858 on PFS for patients with acquired resistance to EGFR-TKIs was from non-T790M subgroup. We consider over-expression of lncRNA BC087858 as a novel mechanism by which acquired resistance to EGFR-TKIs can develop in EGFR-mutant NSCLC patients without T790M mutation. LncRNA BC087858 may stimulate resistance to gefitinib through activation of the PI3K/AKT and MEK/ERK pathways and EMT. Further studies will be required to elucidate the precise mechanisms of lncRNA BC087858-mediated acquired resistance.

## MATERIALS AND METHODS

### Cell lines and cell culture

The human lung adenocarcinoma cell lines PC9 (EGFR exon 19 deletion), H1975 (L858R/T790M), H460 and H23 were obtained from American Type Culture Collection (ATCC, Manassas, USA). The gefitinib-resistant cell line PC9/R and PC9/G2, which has no T790M and MET amplifications, was provided by Shanghai Pulmonary Hospital [21, 24, 36]. All cells were cultured at 37°C in a humidified incubator with 5% CO_2_ in Dulbecco's modified Eagle's medium (DMEM) [Hyclone, Logan, UT, USA] supplemented with 10% fetal bovine serum (FBS) [Sigma Aldrich].

### Tissue samples collection

Seventy eight advanced lung adenocarcinoma tissues were collected from NSCLC patients who had either an exon 19 deletion (19DEL) or an exon 21 point mutation (L858R) in their EGFRs, and were treated with either gefitinib or erlotinib between November 2011 and December 2013, with the written consent of the patients involved and the approval of the Shanghai Pulmonary Hospital Ethics Committee. All patients had either prolonged stable disease (SD) of more than 6 months or a partial response (PR) to EGFR-TKIs therapy and 40 of 78 patients met the established clinical definition of acquired resistance to EGFR-TKIs [37]. Primary resistance to EGFR-TKI was defined as progression on the first imaging evaluation or SD < 6 months after EGFR-TKI treatment in the first setting for patients with NSCLC harboring an activating EGFR mutation. Efficacy data were monitored until the end of December 2015. Of these 78 collected samples, 40 were collected from patients after they developed acquired resistance to EGFR-TKIs (defined as AR group). Other 38 were collected from patients before EGFR-TKIs treatment (defined as BT group).

### Quantitative reverse transcription polymerase chain reaction (qRT-PCR)

Total RNA was extracted from the lung cancer cell lines using TRIzol reagent (TaKaRa, Japan) or from tissue samples using an RNeasy Mini Kit (QIAGEN). The expression of BC087858 in lung cancer cell lines and tissues was measured by qPCR methodology using SYBR Premix Ex Taq (TaKaRa) and an MX3000P instrument. BC087858 primers were designed by Sangon Biotech (China). Glyceraldehyde 3-phosphate dehydrogenase (GAPDH) was used as a control. All experiments were performed in triplicate, and the median of each triplicate set of values was used to calculate relative lncRNA concentrations as follows:

ΔCt (Cycle threshold) = Ct_median lncRNA_ − Ct_median GAPDH_

Fold changes were calculated using 2^−ΔΔCt^ methods.

### Si-RNA transfection

PC9/R, PC9/G2 and H1975 lung cancer cells (2 × 10^5^) were seeded into each well of 6-well plates and incubated overnight, and then transfected with 75nmol/L of small-interfering (si)-BC087858 and a negative control (NC) purchased from RiboBio (Guangzhou, China) that consisted of ribo FECT ™ CP Transfection regent (RiboBio, Guangzhou, China). The target sequence for si-BC087858 was as follows:

Sense strand, 5′ -CCUGGAUCAUCCAGGUCUU dTdT-3′;

Anti-sense strand, 3′ -dTdT GGACCUAGUAGGU CCAGAA-5′.

Forty-eight hours after transfection, the cells were harvested for real-time PCR-Trans-well assay, flow cytometry analysis and western blot analysis.

### Cell proliferation and apoptosis assays

After transfection, the cells were seeded overnight at a density of 5 × 10^3^ cells in 96-well plates in DMEM containing 10% FBS, and then exposed to various concentrations of gefitinib for 72 hours. 10 μLof CCK-8 reagent (Dojingdo Molecular Technology, Japan) was added to the cells for 1hour at 37°C, and the absorbance in each well were measured at 450 nm by an enzyme-labeled instrument. The PC9/R and PC9/G2 cells were seeded in 6-well plates for 24 hours and then transfected with si-BC087858 and the negative control. After gefitinib treatment for 72 hours, the cells were trypsinized, washed twice with PBS, and resuspended in binding buffer. They were then stained with Annexin V/PI (Invitrogen, USA) for 15min in the dark at room temperature, and the cell populations were analyzed by a flow cytometer.

### Trans-well invasion assay

The matrigel (BD Biosciences) was diluted with serum-free DMEM at 1:8, and carefully added 150 μL to the bottom of the trans-well insert (8 μm, BD Biosciences) placed in a 24-well plate without any bubbles. For every group, triplicates were maintained. The trans-well inserts were incubated at 37°C at least 12 h before seeding cells. Cells were detached using 0.025% trypsin-EDTA and washed with PBS, centrifuged and resuspended in serum free DMEM. For each group, 5 × 10^4^ cells were seeded in every trans-well insert, and DMEM supplied with 10% FBS was added in every well of the 24-well plate. After culturing for 24 h, the cells that adhered in the inserts were carefully wiped and fixed with 70% methanol and stained with 0.1% crystal violet. The images were taken with light microscope imaging system (IX73, Olympus, Japan)

### Western blot

Cells were lysed using RIPA protein extraction reagent (Beyotime, Beijing, China) supplemented with phenylmethanesulfonyl fluoride (PMSF) [Riche, CA, USA]. Approximately 25 μg of protein extracts were separated by 10% sodium dodecyl sulfate polyacrylamide gel electrophoresis (SDS-PAGE), transferred onto nitrocellulose membranes (Sigma), and incubated with specific antibodies. An enhanced chemiluminescent (ECL) chromogenic substrate was used to visualize the bands. The blots were developed with a chemiluminescence system, and GAPDH was used as a control. All antibodies were purchased from Abcam (Cambridge, UK).

### Statistical analysis

All statistical analyses were performed using SPSS version 17.0 software (SPSS, Inc., Chicago, IL, USA). Results were presented as the means ± standard deviation (SD) or Standard Error of Mean (SEM) of 3 separate assays. Differences between the different groups were assessed using a *t*-test (two-tailed). Cumulative survival was evaluated using the Kaplan-Meier method, and differences were assessed using the log-rank test. To determine independent prognostic factors, a Cox multivariate regression analysis was used. A *P* value < 0.05 was considered to indicate statistical significance.

## SUPPLEMENTARY MATERIALS FIGURES AND TABLES



## References

[1] Torre LA, Bray F, Siegel RL, Ferlay J, Lortet-Tieulent J, Jemal A (2015). Global cancer statistics, 2012. CA Cancer J Clin.

[2] Torre LA, Siegel RL, Jemal A (2016). Lung Cancer Statistics. Adv Exp Med Biol.

[3] Chen Z, Fillmore CM, Hammerman PS, Kim CF, Wong KK (2014). Non-small-cell lung cancers: a heterogeneous set of diseases. Nat Rev Cancer.

[4] Tan CS, Gilligan D, Pacey S (2015). Treatment approaches for EGFR-inhibitor-resistant patients with non-small-cell lung cancer. Lancet Oncol.

[5] Maemondo M, Inoue A, Kobayashi K, Sugawara S, Oizumi S, Isobe H, Gemma A, Harada M, Yoshizawa H, Kinoshita I, Fujita Y, Okinaga S (2010). Gefitinib or chemotherapy for non-small-cell lung cancer with mutated EGFR. N Engl J Med.

[6] Mok TS, Wu YL, Thongprasert S, Yang CH, Chu DT, Saijo N, Sunpaweravong P, Han B, Margono B, Ichinose Y, Nishiwaki Y, Ohe Y (2009). Gefitinib or carboplatin-paclitaxel in pulmonary adenocarcinoma. N Engl J Med.

[7] Rosell R, Carcereny E, Gervais R, Vergnenegre A, Massuti B, Felip E, Palmero R, Garcia-Gomez R, Pallares C, Sanchez JM, Porta R, Cobo M (2012). Erlotinib versus standard chemotherapy as first-line treatment for European patients with advanced EGFR mutation-positive non-small-cell lung cancer (EURTAC): a multicentre, open-label, randomised phase 3 trial. Lancet Oncol.

[8] Sequist LV, Yang JC, Yamamoto N, O'Byrne K, Hirsh V, Mok T, Geater SL, Orlov S, Tsai CM, Boyer M, Su WC, Bennouna J (2013). Phase III study of afatinib or cisplatin plus pemetrexed in patients with metastatic lung adenocarcinoma with EGFR mutations. J Clin Oncol.

[9] Sequist LV, Waltman BA, Dias-Santagata D, Digumarthy S, Turke AB, Fidias P, Bergethon K, Shaw AT, Gettinger S, Cosper AK, Akhavanfard S, Heist RS (2011). Genotypic and histological evolution of lung cancers acquiring resistance to EGFR inhibitors. Sci Transl Med.

[10] Ohashi K, Maruvka YE, Michor F, Pao W (2013). Epidermal growth factor receptor tyrosine kinase inhibitor-resistant disease. J Clin Oncol.

[11] Chen J, Wang R, Zhang K, Chen LB (2014). Long non-coding RNAs in non-small cell lung cancer as biomarkers and therapeutic targets. J Cell Mol Med.

[12] Esteller M (2011). Non-coding RNAs in human disease. Nat Rev Genet.

[13] Cheetham SW, Gruhl F, Mattick JS, Dinger ME (2013). Long noncoding RNAs and the genetics of cancer. Br J Cancer.

[14] Sang H, Liu H, Xiong P, Zhu M (2015). Long non-coding RNA functions in lung cancer. Tumour Biol.

[15] Cai B, Song XQ, Cai JP, Zhang S (2014). HOTAIR: a cancer-related long non-coding RNA. Neoplasma.

[16] Gutschner T, Hammerle M, Diederichs S (2013). MALAT1—a paradigm for long noncoding RNA function in cancer. J Mol Med (Berl).

[17] Yang Y, Li H, Hou S, Hu B, Liu J, Wang J (2013). The noncoding RNA expression profile and the effect of lncRNA AK126698 on cisplatin resistance in non-small-cell lung cancer cell. PLoS One.

[18] Cheng N, Li X, Zhao C, Ren S, Chen X, Cai W, Zhao M, Zhang Y, Li J, Wang Q, Zhou C (2015). Microarray expression profile of long non-coding RNAs in EGFR-TKIs resistance of human non-small cell lung cancer. Oncol Rep.

[19] Amato KR, Wang S, Tan L, Hastings AK, Song W, Lovly CM, Meador CB, Ye F, Lu P, Balko JM, Colvin DC, Cates JM (2016). EPHA2 Blockade Overcomes Acquired Resistance to EGFR Kinase Inhibitors in Lung Cancer. Cancer Res.

[20] Costa FF (2008). Non-coding RNAs, epigenetics and complexity. Gene.

[21] Li J, Li X, Ren S, Chen X, Zhang Y, Zhou F, Zhao M, Zhao C, Chen X, Cheng N, Zhao Y, Zhou C (2014). miR-200c overexpression is associated with better efficacy of EGFR-TKIs in non-small cell lung cancer patients with EGFR wild-type. Oncotarget.

[22] Li B, Ren S, Li X, Wang Y, Garfield D, Zhou S, Chen X, Su C, Chen M, Kuang P, Gao G, He Y (2014). MiR-21 overexpression is associated with acquired resistance of EGFR-TKI in non-small cell lung cancer. Lung Cancer.

[23] Wang YS, Wang YH, Xia HP, Zhou SW, Schmid-Bindert G, Zhou CC (2012). MicroRNA-214 regulates the acquired resistance to gefitinib via the PTEN/AKT pathway in EGFR-mutant cell lines. Asian Pac J Cancer Prev.

[24] Cheng N, Cai W, Ren S, Li X, Wang Q, Pan H, Zhao M, Li J, Zhang Y, Zhao C, Chen X, Fei K (2015). Long non-coding RNA UCA1 induces non-T790M acquired resistance to EGFR-TKIs by activating the AKT/mTOR pathway in EGFR-mutant non-small cell lung cancer. Oncotarget.

[25] Hampton KK, Craven RJ (2014). Pathways driving the endocytosis of mutant and wild-type EGFR in cancer. Oncoscience.

[26] Rolfo C, Giovannetti E, Hong DS, Bivona T, Raez LE, Bronte G, Buffoni L, Reguart N, Santos ES, Germonpre P, Taron M, Passiglia F (2014). Novel therapeutic strategies for patients with NSCLC that do not respond to treatment with EGFR inhibitors. Cancer Treat Rev.

[27] Miyoshi S, Kato T, Katayama H, Ito R, Mizuno Y, Okura T, Higaki J (2015). A case of EGFR mutant lung adenocarcinoma that acquired resistance to EGFR-tyrosine kinase inhibitors with MET amplification and epithelial-to-mesenchymal transition. Onco Targets Ther.

[28] Soucheray M, Capelletti M, Pulido I, Kuang Y, Paweletz CP, Becker JH, Kikuchi E, Xu C, Patel TB, Al-Shahrour F, Carretero J, Wong KK (2015). Intratumoral Heterogeneity in EGFR-Mutant NSCLC Results in Divergent Resistance Mechanisms in Response to EGFR Tyrosine Kinase Inhibition. Cancer Res.

[29] Witta SE, Gemmill RM, Hirsch FR, Coldren CD, Hedman K, Ravdel L, Helfrich B, Dziadziuszko R, Chan DC, Sugita M, Chan Z, Baron A (2006). Restoring E-cadherin expression increases sensitivity to epidermal growth factor receptor inhibitors in lung cancer cell lines. Cancer Res.

[30] Xia L, Huang W, Tian D, Zhu H, Qi X, Chen Z, Zhang Y, Hu H, Fan D, Nie Y, Wu K (2013). Overexpression of forkhead box C1 promotes tumor metastasis and indicates poor prognosis in hepatocellular carcinoma. Hepatology.

[31] Janne PA, Yang JC, Kim DW, Planchard D, Ohe Y, Ramalingam SS, Ahn MJ, Kim SW, Su WC, Horn L, Haggstrom D, Felip E (2015). AZD9291 in EGFR inhibitor-resistant non-small-cell lung cancer. N Engl J Med.

[32] Sequist LV, Soria JC, Goldman JW, Wakelee HA, Gadgeel SM, Varga A, Papadimitrakopoulou V, Solomon BJ, Oxnard GR, Dziadziuszko R, Aisner DL, Doebele RC (2015). Rociletinib in EGFR-mutated non-small-cell lung cancer. N Engl J Med.

[33] Lan W, Zhang D, Jiang J (2013). The roles of LSD1-mediated epigenetic modifications in maintaining the pluripotency of bladder cancer stem cells. Med Hypotheses.

[34] Ding J, Zhang ZM, Xia Y, Liao GQ, Pan Y, Liu S, Zhang Y, Yan ZS (2013). LSD1-mediated epigenetic modification contributes to proliferation and metastasis of colon cancer. Br J Cancer.

[35] Qi W, Chan H, Teng L, Li L, Chuai S, Zhang R, Zeng J, Li M, Fan H, Lin Y, Gu J, Ardayfio O (2012). Selective inhibition of Ezh2 by a small molecule inhibitor blocks tumor cells proliferation. Proc Natl Acad Sci U S A.

[36] Ju L, Zhou C, Li W, Yan L (2010). Integrin beta1 over-expression associates with resistance to tyrosine kinase inhibitor gefitinib in non-small cell lung cancer. J Cell Biochem.

[37] Gainor JF, Shaw AT (2013). Emerging paradigms in the development of resistance to tyrosine kinase inhibitors in lung cancer. J Clin Oncol.

