# Supplementary material for: Long non-coding RNA BC087858 induces non-T790M mutation acquired resistance to EGFR-TKIs by activating PI3K/AKT and MEK/ERK pathways and EMT in non-small-cell lung cancer
Source: Oncotarget. 2016 Jul 9;7(31):49948–60. doi: 10.18632/oncotarget.10521 (PMC5226560; doi:10.18632/oncotarget.10521)
Supplement: Supplementary file 1 [file oncotarget-07-49948-s001.pdf]

Long non-coding RNA BC087858 induces non-T790M mutation acquired resistance to EGFR-TKIs by activating PI3K/AKT and MEK/ERK pathways and EMT in non-small-cell lung cancer

Supplementary Materials

Supplementary Table 1: Progression free survival (PFS) cut-off point analysis of BC087858

Cut-off point		PFS		
		Log-Rank	HR(95%CI)	*P*
10	0.004	0.1495	0.763 (0.1942–3.001)	0.699
15	0.007	2.296	0.424 (0.1401–1.286)	0.130
20	0.012	2.296	0.424 (0.1401–1.286)	0.130
25	0.020	2.332	0.423 (0.1399–1.277)	0.127
30	0.028	2.535	0.438 (0.1588–1.21)	0.111
35	0.044	1.79	0.507 (0.1871–1.372)	0.507
40	0.049	0.397	0.73 (0.2745–1.942)	0.528
45	0.053	2.442	0.436 (0.1536–1.235)	0.118
50	0.057	3.79	0.352 (0.1232–1.007)	0.052
55	0.064	2.527	0.425 (0.1479–1.221)	0.112
60	0.072	4.658	0.279 (0.08751–0.8893)	0.031
65	0.103	3.397	0.328 (0.09996–1.073)	0.065
70	0.142	5.271	0.232 (0.06637–0.8073)	0.021
75	0.175	2.826	0.342 (0.09760–1.195)	0.093
80	0.230	3.521	0.207 (0.03994–1.073)	0.061
85	0.379	1.786	0.303 (0.05256–1.746)	0.181
90	0.702	5.344	0.0294 (0.001481–0.5848)	0.021
